# Passive Sampling for Indoor and Outdoor Exposures to Chlorpyrifos, Azinphos-Methyl, and Oxygen Analogs in a Rural Agricultural Community

**DOI:** 10.1289/EHP425

**Published:** 2016-08-12

**Authors:** Jenna L. Gibbs, Michael G. Yost, Maria Negrete, Richard A. Fenske

**Affiliations:** 1 Department of Occupational and Environmental Health, University of Iowa College of Public Health, Iowa City, Iowa, USA; 2 Department of Environmental and Occupational Health Sciences, University of Washington School of Public Health, Seattle, Washington, USA

## Abstract

**Background:**

Recent studies have highlighted the increased potency of oxygen analogs of organophosphorus pesticides. These pesticides and oxygen analogs have previously been identified in the atmosphere following spray applications in the states of California and Washington.

**Objectives:**

We used two passive sampling methods to measure levels of the ollowing organophosphorus pesticides: chlorpyrifos, azinphos-methyl, and their oxygen analogs at 14 farmworker and 9 non-farmworker households in an agricultural region of central Washington State in 2011.

**Methods:**

The passive methods included polyurethane foam passive air samplers deployed outdoors and indoors and polypropylene deposition plates deployed indoors. We collected cumulative monthly samples during the pesticide application seasons and during the winter season as a control.

**Results:**

Monthly outdoor air concentrations ranged from 9.2 to 199 ng/m^3^ for chlorpyrifos, 0.03 to 20 ng/m^3^ for chlorpyrifos-oxon, < LOD (limit of detection) to 7.3 ng/m^3^ for azinphos-methyl, and < LOD to 0.8 ng/m^3^ for azinphos-methyl-oxon. Samples from proximal households (≤ 250 m) had significantly higher outdoor air concentrations of chlorpyrifos, chlorpyrifos-oxon, and azinphos-methyl than did samples from nonproximal households (*p* ≤ 0.02). Overall, indoor air concentrations were lower than outdoors. For example, all outdoor air samples for chlorpyrifos and 97% of samples for azinphos-methyl were > LOD. Indoors, only 78% of air samples for chlorpyrifos and 35% of samples for azinphos-methyl were > LOD. Samples from farmworker households had higher indoor air concentrations of both pesticides than did samples from non-farmworker households. Mean indoor and outdoor air concentration ratios for chlorpyrifos and azinphos-methyl were 0.17 and 0.44, respectively.

**Conclusions:**

We identified higher levels in air and on surfaces at both proximal and farmworker households. Our findings further confirm the presence of pesticides and their oxygen analogs in air and highlight their potential for infiltration of indoor living environments.

**Citation:**

Gibbs JL, Yost MG, Negrete M, Fenske RA. 2017. Passive sampling for indoor and outdoor exposures to chlorpyrifos, azinphos-methyl, and oxygen analogs in a rural agricultural community. Environ Health Perspect 125:333–341; http://dx.doi.org/10.1289/EHP425

## Introduction

### Organophosphorus Pesticides and Oxygen Analogs

**Figure 1:**
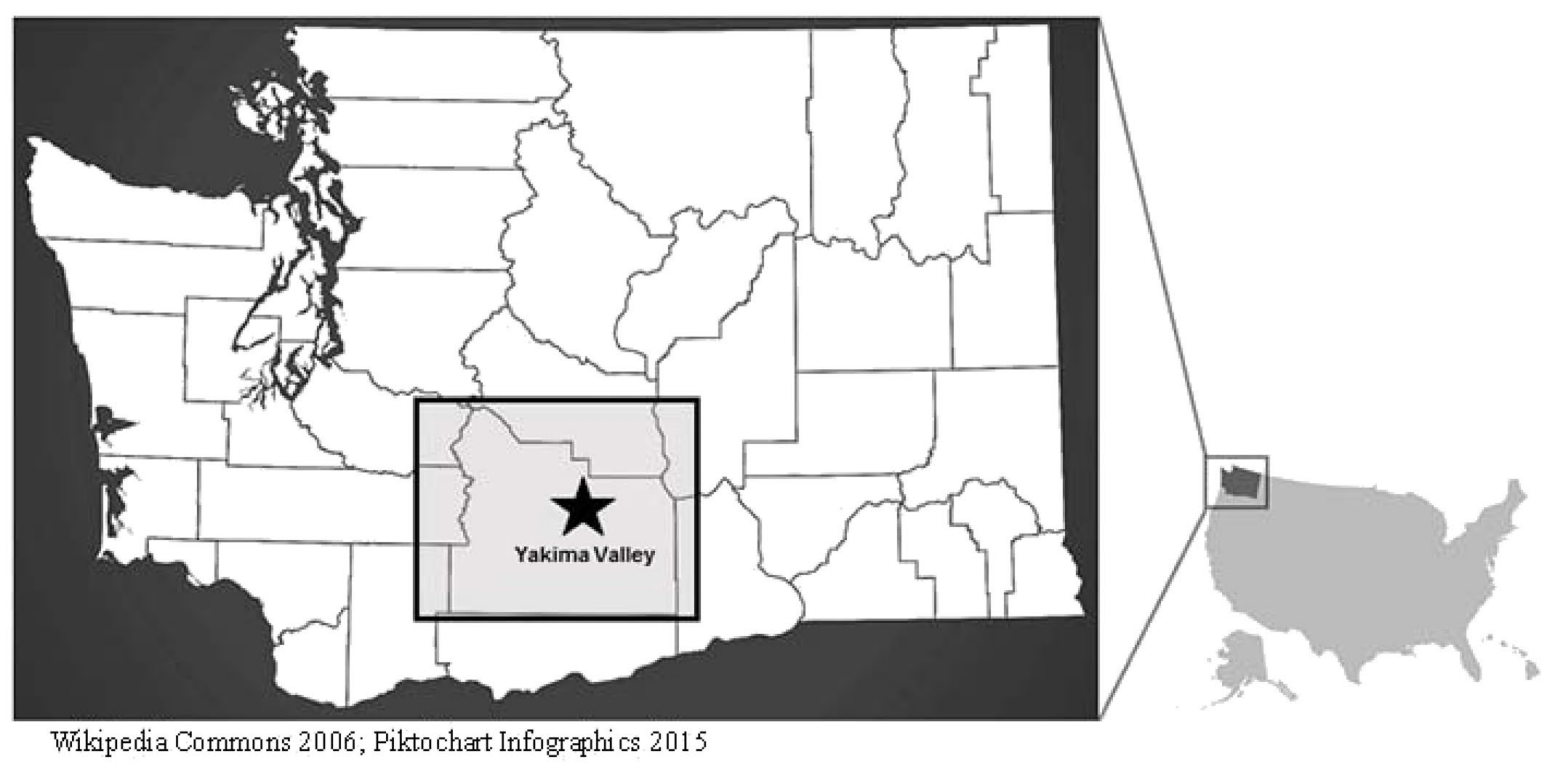
Map of Yakima Valley, Washington State study region.

In the Yakima Valley region of Washington State, there are more than a thousand orchards (e.g., apples, pears, cherries) covering over 100,000 acres. Washington is the lead producer of apples and cherries in the United States, and 10–12 billion apples are picked each year (USDA 2009) (Figure 1 shows a map of the region). The region is also home to many farmworker families and more than half of the population is Hispanic/Latino (U.S. Census Bureau Geography Division 2010). Most of this population is involved in tree fruit production—harvesting, pruning, thinning, and applying agricultural chemicals (Thompson et al. 2008). In 2011, chlorpyrifos (CPF) and azinphos-methyl (AZM) were some of the most commonly applied organophosphorus (OP) pesticides in tree fruit and vegetable production (Baker and Stone 2015). Both pesticides are often applied in aerosolized form to tree fruits using a large sprayer attached to a tractor. In 2012, the U.S. Environmental Protection Agency (EPA) banned the use of AZM in apple production. Prior to the ban, when this study was conducted, AZM and CPF were commonly sprayed with application rates averaging 0.5 kg/acre and 1 kg/acre active ingredient, respectively (USDA 2008).

The use of OP pesticides in Yakima Valley has long been a health concern of local residents to due to potential human exposures resulting from off target volatilization and drift. In 2008, the state of Washington funded a study to examine off-target movement of OP pesticides and potential risk to bystanders (Fenske et al. 2009). In the 2009 study, CPF, AZM, and their oxygen analogs were identified in the outdoor air of the surrounding agricultural communities, indicating direct atmospheric transformation. Other studies have also reported these compounds in air (Armstrong et al. 2013b; CARB 1998; CDPR 2006, 2009).

Toxicology studies have focused on the relative potency of combined OP pesticides and their oxygen analogs in animal models (Costa et al. 2005), and acknowledge transformation to the oxygen analog *in vivo* as a metabolic product through breakdown mechanisms involving cytochrome p450 enzymes. Chlorpyrifos-oxon (CPF-O) is poses a special risk for genetically susceptible individuals who have lower levels of the paraoxonase (PON-1^–/–^) enzyme (Shih et al. 1998), and children may be susceptible to the CPF-O due to differences in their metabolic functioning during development (Barr et al. 2004; Costa et al. 2005). Therefore, it is important to consider the presence of oxygen analogs in the air when measuring human exposure.

In several studies over a decade ago, we found that levels of OP pesticide metabolites in the urine of farmworker children were significantly higher than the levels in the urine of non-farmworker children in the same region (Loewenherz et al. 1997; Lu et al. 2000): These relatively high levels were later confirmed by comparison with national biomonitoring data (Fenske et al. 2005). We also found that pesticide levels in household dust (including AZM and CPF) were higher in farmworker homes than in non-farmworker homes in the same region (Lu et al. 2000; Fenske et al. 2002).

CPF and AZM are both semivolatile compounds, and they exist as both vapor and particle-bound forms in air. This phase-partitioning is highly dependent on a combination of the timing of pesticide application and meteorological factors (Howard 1991). Both compounds can persist for days to weeks outdoors, and for several months indoors (Lewis 2005; Wauchope et al. 1992). There is very little scientific data regarding the long-term atmospheric transport of CPF, AZM, CPF-O, and azinphos-methyl-oxon (AZM-O), and even less is known about their ability to infiltrate indoor environments.

### Passive Sampling for Pesticides

To date, many studies have focused on short and long term human health outcomes associated with OP pesticides (Roberts et al. 2012; Quandt et al. 2006), although very few have incorporated long-term air and surface exposure measurements for OP pesticides and oxygen analogs due to the high costs and invasive procedures associated with residential sampling. The oxygen analogs (CPF-O and AZM-O) are relatively new phenomena, and, to our knowledge, no studies have measured them in residences.

Although active air sampling is useful for examining daily fluctuations or collecting a personal sample over the course of a work shift, it involves frequent collection of sampling media, uses electricity, and requires space for the sampling pumps. In a previous study (Armstrong et al. 2013a), we identified artificial transformation from CPF to CPF-O during active air sampling with OVS/XAD-2 tubes (NIOSH 1994) in a controlled laboratory environment. In response, we developed a polyurethane foam (PUF) passive air sampling (PAS) method that was able to sample for OP pesticides and their oxygen analogs at rates similar to active air sampling at 2 L/min (Armstrong et al. 2014b). CPF and AZM are both suitable for passive air sampling because they have ideal chemical properties, including octanol-air partition coefficients (log K_OA_ values) that fall somewhere between polychlorinated biphenyls (PCBs) and polybrominated diphenyl ethers (PBDEs) (Table 1).

**Table 1:** Chemical properties of CPF, AZM, and suitability for passive sampling with PUF-PAS.

Chemical	Mol. weight	Log K_ow_	Log K_OA_^*a*^	Solubility (mg/L)	Henry’s Constant^*b*^ (atm m^3^/mole)	Vapor pressure (mmHg) (25°C)	Volatility
Chlorpyrifos	350.59	4.27	8.36	0.39	6.76E-01	1.23E-05	Semi-volatile
Azinphos methyl	317.32	3.38	11.34	14	5.70E-06	3.80E-04	Semi-volatile
Note: PUF-PAS, polyurethane foam passive air sampling. Since data on oxygen analogs are limited, the analog compound was assumed to have chemical properties similar to the parent compound. Data on chemical properties are from PubChem CID 2016, 2268, and 2730 (NCBI 2015a, 2015b). ^***a***^Log K_OA_ (octanol air partition coefficient) is calculated from the log K_OW_ (octanol water partition coefficient) using the ideal gas constant and Henry’s Constant value (Meylan and Howard 2005). ^***b***^Henry’s Constant values > 10^–8^ and log K_OA_ values 7–13 indicate that the compound is ideal for passive sampling with PUF.							

Another common indoor passive sampling method involves deposition plates to collect settled particulate. Since the deposition method collects larger diameter particles (as opposed to gases), it is a useful measure of particle-bound phase and dust settling. Deposition plates for indoor OP pesticides have been used in previous studies using polyethylene, chromatography paper, and double-layer gauze pads backed by aluminum foil (Keenan et al. 2010; Lu and Fenske 1998).

Our overall aim of this study was to use passive sampling methodologies to measure airborne and surface deposition levels of CPF, AZM, CPF-O, and AZM-O outside and inside of households in a rural agricultural region. Our secondary aim was to compare the levels between proximal and non-proximal and between farmworker and non-farmworker households to determine if certain groups were at higher risk of exposure.

## Study Methods

### Sampling Plan

**Figure 2:**
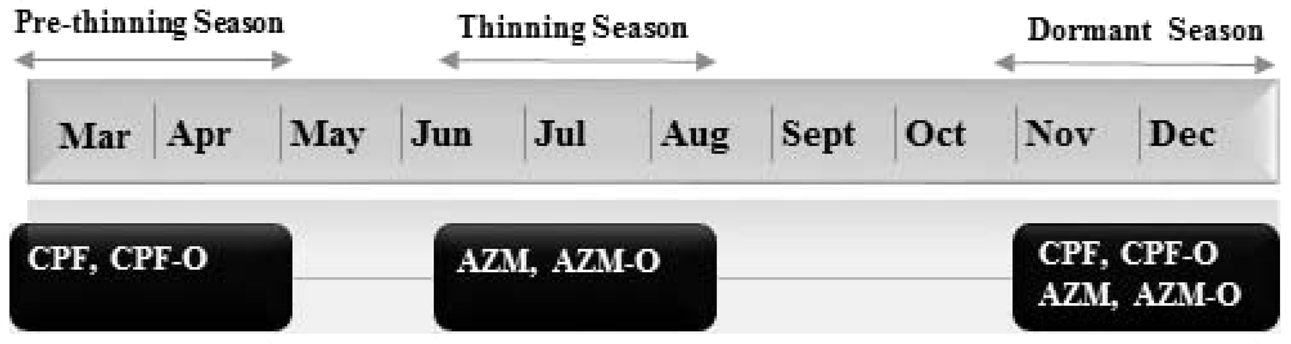
Sampling time-line. Sampling occurred in 2011 during the spring application season for CPF and CPF-O, during the summer application season for AZM and AZM-O, and during the winter dormancy season for CPF, CPF-O, AZM, and AZM-O.

We conducted the residential sampling during three seasons in 2011: *a*) the spring pre-thinning season for CPF and CPF-O, *b*) the summer thinning season for AZM and AZM-O, and *c*) winter dormancy season for CPF, CPF-O, AZM, and AZM-O (Figure 2 shows timeline). The pre-thinning application, thinning application, and winter dormancy seasons were defined using CPF and AZM product information from Washington State University’s Decision Aid System (https://www.decisionaid.systems/), which uses meteorologic and entomologic data to predict optimal pesticide application times for tree fruit producers. In addition, we contacted local Washington State agricultural extension agents to inform us about field activity.

Twenty-three sampling locations were selected *a priori* to be equally grouped as proximal (≤ 250 m of any nearest tree fruit field) and nonproximal (> 250 m). Of these, 20 participants were recruited from households enrolled with the Para Niños Saludables project. This is a community-based research project led by researchers at the Fred Hutchinson Cancer Research Center involving a cohort of 60 farmworker and 40 non-farmworker families. We defined farmworker households as having one or more current farmworkers (temporary or full time), and non-farmworker households as having no farmworkers living in the household (employment status was obtained from the 2011 Para Niños Saludables household survey). Details about the project and population have been previously reported by Thompson et al. (2008). Overall, there were 6 proximal farmworker, 2 proximal non-farmworker, 7 nonproximal farmworker, and 5 nonproximal and non-farmworker households (see Table S1).

The three remaining sampling locations were outdoor community air monitoring sites (managed by the Yakama Nation Environmental Protection Program) within 100 m of the nearest Para Niños Saludables residence. During the study, these community locations were required to support replicate sampling and side-by-side comparisons with the active air sampling methods for quality assurance purposes. In addition, for data analysis, at these locations the outdoor measurements were used as surrogates for the nearest household. We used proximity and farmworker employment data from the nearest household and checked to make sure surrogate community locations and participant residences were equidistant from tree fruit fields. One of the community sites was located in a rural area near a proximal farmworker household. The other two community sites were urban, near nonproximal non-farmworker households.

We plotted all locations in ArcGIS (version 10.0; ESRI, Redlands, CA) using GPS coordinates collected with a GPS Map 60CS handheld unit (Garmin, Inc., Olathe, KS). We identified tree fruit fields using a Cropland Data Layer from USDA CropScape. The Cropland Data Layer is a geo-referenced, crop-specific land cover data layer created annually using satellite imagery and extensive agricultural ground cover (USDA-NASS 2012). We checked to make sure surrogate community locations and participant residences were equidistant from tree fruit fields. Since such a large portion of the rural community was involved in agriculture, it was challenging to identify non-farmworker households that were also proximal (only two households were recruited).

We collected a total of 66 outdoor air samples (CPF and CPF-O, *n* = 36; AZM and AZM-O, *n* = 30), and 53 indoor air and surface deposition samples (CPF and CPF-O, *n* = 27; AZM and AZM-O, *n* = 26) during the application seasons. These numbers include duplicate and triplicate samples deployed in the same location at the same time for quality control purposes (see Table S1 for description of replicate samples). We deployed 7 outdoor air samples and 7 indoor air and surface deposition samples at six locations during the winter dormant season as a control. These winter locations were chosen for optimal geospatial distribution across the region.

This study followed protocols approved by the Fred Hutchinson Cancer Research Center Institutional Review Board. Written informed consent (in Spanish or English) was obtained for all households in the study. A field industrial hygienist scheduled a meeting with the promotora and the household members to set up the samplers. Outdoors, we placed the PUF-PAS away from children’s play areas, buffers (≥ 8 m from trees and buildings), livestock, and other high foot traffic areas. Indoors, we placed the PUF-PAS and deposition plates in the living room or kitchen to capture an area of the house where family members spend a large amount of time. This location was placed ≥ 1 m high on a shelf or desk to minimize interference or contact with other surfaces (e.g., walls, windows, doors). Monthly sampling periods ranged from 24 to 32 days. At each household, outdoor and indoor samples were deployed and collected on the same day. During the time of collection, we obtained qualitative participant feedback about the passive samplers.

### Sampling Materials

The PUF-PAS device uses properties of atmospheric diffusion to collect contaminants without the use of a pump and sampling rate is controlled by diffusivity (Hourani and Underhill 1988; Shoeib and Harner 2002). The PUF-PAS method for measurement of OP pesticides and oxygen analogs was previously tested in both laboratory and field environments by Armstrong et al. (2014b) using depuration compounds and side-by-side comparisons with more traditional active sampling methods (U.S. EPA 1999). We derived average air concentration (*C_air_*, ng/m^3^) from the sampling rate (*R_PUF-PAS_*, m^3^/day) and the mass of pesticide on the matrix (*M_pas_*, ng), where *t* = time in days (Equation 1):

*C_air_* = *M_pas_*/(*R_PUF-PAS_* × *t*) [1]

Prior to deployment, we spiked each outdoor PUF-PAS with depuration compounds [210 ng of CPF-methyl-D_6_ (99%, 100 μg/mL in acetonitrile; EQ Laboratories, Atlanta, GA) and 450 ng of AZ-ethyl-D_10_ (98.5%, 1,000 μg/mL in toluene; EQ Laboratories)] with a 50 μL Hamilton positive displacement syringe. Depuration compounds were not used indoors to ensure safety of residents. We calculated outdoor sampling rates, or R_PUF-PAS_, using the loss of depuration compounds from the PUF matrix and by calibration with side-by-side active air sampling (AAS). All procedures and calculation of sampling rates have been described by Armstrong et al. (2014b).

Outdoors, we placed the PUF-PAS disk (Tisch Environmental, 14 cm in diameter, 1.3 cm thick, surface area 370 cm^2^) in a stainless steel, domed chamber (22 cm diameter) to protect from wind, precipitation, and sunlight (Shoeib and Harner 2002; Schuster et al. 2012; Tuduri et al. 2006). Air was allowed to flow over the PUF disks through a 1.5 cm gap between chamber encasements. The sampling housing was hooked to a steel sampling mast at 1.5 m height. After collection, the PUF sample media was sealed in a glass Petri dish, and stored in a –20°C freezer.

Indoors, the shape and surface area of the PUF-PAS was cylindrical (7 × 3 cm diameter, 74 cm^2^ surface area), and similar to the mini-PUF introduced by Bohlin et al. (2010). We hung the cylinder from a 22 cm tall free-standing hook. Next to the indoor PUF-PAS, a small surface deposition plate consisting of a Petri dish (6 cm diameter, 89000-300 VWR) lined with a polypropylene (PP) filter (5 μm pore, 17.3 cm^2^ surface area, Whatman) collected deposited particulate. A temperature logger (LogTag TRIX-8) was placed near both passive sampling devices. After collection we wrapped indoor PUF-PAS cylinders in aluminum foil and stored them in zipper-sealed bags, covered and sealed deposition plates, and stored both sample types similarly to outdoor samples. Indoor air concentrations (*C_air_*, ng/m^3^) were derived using the same calculation (Equation 1) as for outdoors. Since depuration compounds were not used indoors, indoor sampling rates (*R_PUF-PAS_*, m^3^/day) were estimated using the *K_A_* (air-side mass transfer coefficient) and the surface area (*S_area_*) of the indoor PUF cylinder (74 cm^2^) (Equation 2). We determined *K_A_* from the average loss of depuration compounds in previous laboratory tests at 25°C (Armstrong et al. 2014b). K_A_ was adjusted for average indoor temperatures recorded by the indoor temperature logger. The calculation, below, has also been described by Shoeib and Harner (2002) (Equation 2):

*Indoor R_PUF-PAS_* = *K_A_* × *S_area_* [2]

For the surface deposition samples, we divided the mass of pesticide (*M_pp_*) by the surface area (*S_area_*) to obtain a mass loading (*S_load_*, ng/cm^2^) (Butte and Heinzow 2002) (Equation 3):

*S_load_* = *M_pp_*/(*S_area_*) [3]

### Chemical Analysis

Preparation and storage of PUF-PAS matrices followed similar procedures used in other studies (Bohlin et al. 2010; Shoeib and Harner 2002). We rinsed Petri dishes and aluminum foil with solvent during extraction. PUF-PAS and PP filter matrices were sonicated for 1.5 hr at room temperature (20–23°C) in 10–50 mL acetonitrile solution containing stable-isotope labeled internal standards and then evaporated to 1.5 mL. Large particulate was filtered with a PTFE syringe filter (13 mm, 0.2 μm porosity). Sample analysis was conducted using the liquid chromatography tandem mass spectrometry (LC-MS/MS) method with internal standards (Armstrong et al. 2014a, 2014b). Instrument limits of detection (LOD) were 1 ng/sample for CPF and CPF-O, and 1 ng/sample AZM, and 5 ng/sample for AZM-O. The instrument LOD for all depuration compounds was 1 ng/sample. After accounting for the volume of PUF-PAS and surface area of deposition plates, this corresponded to PUF-PAS method Limit of Quantification (LOQ) ranging from 0.01 to 0.02 ng/m^3^ for CPF/CPF-O and 0.02 to 0.03 ng/m^3^ for AZM/AZM-O; and a surface deposition plate method LOQ of 0.03 ng/cm^2^ for CPF/CPF-O and 0.17 ng/cm^2^ for AZM/AZM-O.

Coefficients of variation (CV) were ≤ 19% for CPF, ≤ 9% for CPF-O, ≤ 37% for AZM, and ≤ 10% for AZM-O in outdoor air samples; and ≤ 6% for CPF indoor air samples. For the surface deposition plates, CVs were ≤ 15% for CPF and all replicate samples for AZM were below the LOD. We were unable to calculate CVs for AZM, CPF-O, and AZM-O from indoor air samples and surface deposition plates because replicate samples were < LOD. All field blanks were below the LOD for CPF, CPF-O, AZM, and AZM-O. Storage stability and spike fortification recovery results were 80–120% for all measured compounds.

### Data Analysis

For air samples below the LOD, we assigned a substitute value for M_pas_ and M_pp_ by taking LOD divided by the square root of 2 and divided by the effective air sampling volume (Equation 1 and 2), or surface area (Equation 3), respectively. We calculated the mean, standard deviation, and range for outdoor and indoor air concentrations (ng/m^3^) and indoor surface deposition (ng/m^2^) among household types (i.e., proximal farmworker, proximal non-farmworker, non-proximal farmworker, and non-proximal non-farmworker). We compared group results using a 2-way non-parametric Friedman test (α = 0.10), which is similar to a parametric repeated measure ANOVA (Zimmerman and Zumbo 1993). Next, we compared outdoor and indoor air concentrations and indoor surface deposition in proximal vs. non-proximal and farmworker vs. non-farmworker variables using a non-parametric Kruskal–Wallis one way ANOVA test (α = 0.05). Replicate samples were included in these calculations.

Since both outdoor and indoor air samples were collected simultaneously, we calculated indoor/outdoor mean ratios for each household by dividing the indoor air concentration by the outdoor air concentration (the mean of replicate samples was used when necessary). We then calculated the mean of these ratios by household type. A ratio greater than 1 indicates higher indoor pesticide concentrations, whereas a ratio less than 1 indicates higher outdoor pesticide concentrations. We compared indoor and outdoor mean ratios in proximal versus non-proximal and farmworker versus non-farmworker households using a non-parametric Kruskal–Wallis one way ANOVA test (α = 0.05).

Finally, we calculated the Spearman’s correlation coefficient (*R*_s_) between air concentrations and surface deposition indoors. Since replicate sampling can influence correlation results, the mean of replicate samples was used for this calculation. All statistical calculations were performed in Stata (version 11.2; College Station, TX). We did not compare group results of outdoor and indoor air concentrations or indoor surface deposition for the winter samples due to limitations of small sample size. We deployed samples in the winter to primarily test for presence of OP pesticides during a dormant season.

## Results

### Outdoor Air Concentrations

We present the results of outdoor air concentrations by household type in Table 2. All air samples yielded detectable CPF and CPF-O. During the spring, cumulative residential air concentrations of CPF ranged from 9.2 to 199 ng/m^3^, and concentrations of CPF-O ranged from 0.03 to 20 ng/m^3^. We identified the highest levels of CPF (3 of 36 samples > 100 ng/m^3^) at proximal farmworker households within 100 m of apple, peach, corn, or wheat fields. We identified the highest levels of CPF-O (3 of 36 samples > 13 ng/m^3^) at both proximal farmworker and proximal non-farmworker households within 100 m of apple, peach, corn, or wheat fields.

**Table 2:** Summary of outdoor and indoor air concentrations (ng/m^3^).

Sample type	Proximal farmworker			Proximal non-farmworker			Non-proximal farmworker			Non-proximal non-farmworker			Total
	*n* (*k*)	*n* < LOD	Mean ± Std (min, max)	*n* (*k*)	*n* < LOD	Mean ± Std (min, max)	*n* (*k*)	*n* < LOD	Mean ± Std (min, max)	*n* (*k*)	*n* < LOD	Mean ± Std (min, max)	*n* (*k*)
Outdoor air
Spring
CPF	12 (7)	0	72 ± 60^*a*^ (20 – 199)	6 (2)	0	31 ± 15^*a*^ (12 – 44)	9 (7)	0	23 ± 13^*a*^ (9.2 – 42)	9 (7)	0	11 ± 6.2^*a*^ (9.7 – 19)	36 (23)
CPF-O		0	10 ± 5.5^*a*^ (3.0 – 20)		0	4.5 ± 4.2^*a*^ (2.7 – 15)		0	3.2 ± 3.1^*a*^ (0.03 – 7.9)		0	2.5 ± 1.5^*a*^ (2.2 – 4.3)	
Summer
AZM	9 (7)	0	2.4 ± 2.6^*a*^ (0.4 – 7.3)	4 (2)	0	1.2 ± 0.4^*a*^ (0.7 – 1.6)	8 (7)	0	0.3 ± 0.1^*a*^ (0.1 – 0.7)	9 (7)	1	0.3 ± 0.2^*a*^ (< LOD – 0.6)	30 (23)
AZM-O		4	0.1 ± 0.3 < LOD – 0.8		2	0.03 ± 0.1 (< LOD – 0.1)		8^*b*^	< LOD NA		6^*b*^	0.02 ± 0.03 (< LOD – 0.05)	
Winter^*c*^
CPF	3 (2)	0	3.5 ± 2.7 (0.6 – 5.8)	1 (1)	1	< LOD (NA)	2 (2)	1	0.3 ± 0.4 (< LOD – 0.6)	1 (1)	1	< LOD (NA)	7 (6)
CPF-O		0	0.2 ± 0.2 (0.02 – 0.4)		1	< LOD (NA)		1	0.02 ± 0.01 (< LOD – 0.02)		1	< LOD (NA)	
AZM		3^*b*^	< LOD (NA)		1	< LOD (NA)		2	< LOD (NA)		1	< LOD (NA)	
AZM-O		3^*b*^	< LOD (NA)		1	< LOD (NA)		2	< LOD (NA)		1	< LOD (NA)	
Indoor air
Spring
CPF	8 (6)	1	7.9 ± 6.8 (< LOD – 18)	4 (2)	1	0.5 ± 0.4 (< LOD – 0.8)	8 (7)	2	3.5 ± 3.8 (< LOD – 9.2)	7 (5)	2	0.6 ± 1.8 (< LOD – 0.6)	27 (20)
CPF-O		5^*b*^	0.03 ± 0.1 (< LOD – 0.6)		3^*b*^	0.01 ± 0.01 (< LOD – 0.01)		6^*b*^	0.01 ± 0.1 (< LOD – 0.2)		6^*b*^	0.1 ± 0.2 (< LOD – 0.1)	
Summer
AZM	8 (6)	3	0.1 ± 0.1 (< LOD – 0.2)	3 (2)	3^*b*^	< LOD (NA)	7 (7)	3	0.3 ± 0.2 (< LOD – 0.8)	8 (5)	8^*b*^	< LOD (NA)	26 (20)
AZM-O		6^*b*^	0.10 ± 0.04 (< LOD – 0.3)		3^*b*^	< LOD (NA)		6	0.02 ± 0.3 (< LOD – 0.06)		8^*b*^	< LOD (NA)	
Winter^*c*^
CPF	3 (2)	0	0.2 ± 0.5 (0.02 – 0.9)	1 (1)	1	< LOD (NA)	2 (2)	2	< LOD (NA)	1 (1)	1	< LOD (NA)	7 (6)
CPF-O		3^*b*^	< LOD (NA)		1	< LOD (NA)		2	< LOD (NA)		1	< LOD (NA)	
AZM		3	< LOD (NA)			< LOD (NA)		2	< LOD (NA)		1	< LOD (NA)	
AZM-O		3	< LOD (NA)			< LOD (NA)		2	< LOD (NA)		1	< LOD (NA)	
Note: *k, *number of locations; LOD, limit of detection; NA, not available; *n*, number of samples; *n* < LOD, number of samples < LOD; max, maximum; min, minimum; Std, standard deviation; ^***a***^*p* < 0.1 for differences across all household types (2-way Friedman’s Test). ^***b***^Count (*n* < LOD) includes replicate samples that were < LOD. ^***c***^Winter was a dormant season, therefore a limited number of samples were collected.													

Although 29 of 30 (97%) air samples yielded detectable AZM, only 10 of 30 (33%) samples had detectable AZM-O. During the summer, cumulative air concentrations of AZM and AZM-O were lower than for CPF and CPF-O. Air concentrations of AZM ranged from < LOD to 7.3 ng/m^3^ and AZM-O ranged from < LOD to 0.8 ng/m^3^. We identified the highest levels of AZM (3 of 30 samples > 4 ng/m^3^) and AZM-O (3 of 30 samples > 0.3 ng/m^3^) at proximal farmworker households within 200 m of apple, peach, and cherry fields.

There were significant differences in outdoor air concentrations of CPF, CPF-O, and AZM between proximal-farmworker, proximal non-farmworker, non-proximal farmworker, and non-proximal non-farmworker households (Table 2, 2-way Friedman’s test, *p* < 0.10). Proximal households had higher mean outdoor air concentrations of CPF, CPF-O, and AZM than non-proximal households; and farmworker households also had significantly higher mean outdoor air concentrations of CPF and CPF-O than non-farmworker households (Table 3, Kruskal–Wallis test, *p* < 0.05).

**Table 3:** Comparisons between proximal versus non-proximal and farmworker versus non-farmworker for outdoor air concentrations (ng/m^3^), indoor air concentrations (ng/m^3^), and surface deposition (ng/cm^2^).

Sample type	Proximal		Non-proximal		*p*-Value^*a*^	Farmworker		Non-farmworker		*p*-Value^*a*^
	*n*	Mean ± Std	*n*	Mean ± Std		*n*	Mean ± Std	*n*	Mean ± Std	
Outdoor air (ng/m^3^)
Spring
CPF	18	53.4 ± 53.9	18	18.2 ± 10.1	0.02	21	48.2 ± 50.9	15	17.7 ± 10.4	0.01
CPF-O		7.5 ± 4.3		3.1 ± 2.3	0.01		5.8 ± 4.3		4.6 ± 3.6	0.01
Summer
AZM	13	2.1 ± 2.2	17	0.3 ± 0.2	< 0.001	17	1.4 ± 2.1	13	0.65 ± 0.5	0.18
AZM-O		0.2 ± 0.3		0.02 ± 0.02^*b*^	0.69		0.1 ± 0.2		0.03 ± 0.03^*b*^	0.48
Indoor air (ng/m^3^)
Spring
CPF	12	6.3 ± 5.9	17	1.4 ± 3.0	0.03	16	5.4 ± 5.5	11	0.2 ± 0.3	0.01
CPF-O		0.1 ± 0.2^*b*^		0.02 ± 0.1^*b*^	0.96		0.1 ± 0.2^*b*^		0.01 ± 0.0^*b*^	0.20
Summer
AZM	11	0.04 ± 0.1^*b*^	15	0.1 ± 0.2^*b*^	0.82	15	0.1 ± 0.2^*b*^	11	0.03 ± 0.1^*b*^	0.12
AZM-O		0.03 ± 0.1^*b*^		0.01 ± 0.01^*b*^	NA		0.03 ± 0.1^*b*^		< LOD	NA
Indoor surface (ng/cm^2^)
Spring
CPF	12	1.2 ± 2.2^*b*^	15	0.2 ± 0.5^*b*^	0.01	16	1.0 ± 1.9^*b*^	11	0.1 ± 0.1^*b*^	0.37
CPF-O		0.06 ± 0.1^*b*^		0.01 ± 0.05^*b*^	NA		0.06 ± 0.1^*b*^		< LOD	NA
Summer
AZM	11	0.3 ± 0.5^*b*^	15	0.2 ± 0.2^*b*^	0.03	16	0.4 ± 0.4^*b*^	10	0.04 ± 0.05^*b*^	0.09
AZM-O		0.01 ± 0.01^*b*^		< LOD	NA		< LOD		< LOD	NA
Note: Winter samples were not used in the comparisons due to limited sample size. LOD, limit of detection; NA, not available; *n*, number of samples. ^***a***^*p* < 0.05 for differences between groups (Kruskal–Wallis test). ^***b***^For these samples, the calculated mean and standard deviations include substituted values < LOD.										

### Indoor Air Concentrations

We also present the results of indoor air concentrations by household type in Table 2. Overall, cumulative indoor air concentrations were lower than outdoor concentrations. For example, 21 of 27 (78%) indoor air samples yielded detectable levels of CPF, and only 7 of 27 (26%) had detectable levels of CPF-O. During the spring, indoor air concentrations of CPF ranged from < LOD to 18 ng/m^3^, and all concentrations of CPF-O were ≤ 0.6 ng/m^3^. We identified the highest levels of indoor CPF (4 of 27 samples > 9 ng/m^3^) in proximal and non-proximal farmworker households. Overall, farmworker households had higher indoor air concentrations of CPF than non-farmworker households (Table 3, Kruskal–Wallis test, *p* < 0.05).

During the summer, indoor air concentrations of AZM were lower than CPF, ranging from < LOD to 0.8 ng/m^3^ (Table 2). For example, 9 of 26 (35%) indoor air samples yielded detectable levels of AZM, and only 3 of 26 (12%) had detectable levels of AZM-O. There were no significant differences in indoor air concentrations of AZM or AZM-O between farmworker and non-farmworker and proximal and non-proximal households (Table 3). We identified the highest levels of indoor AZM (2 of 26 samples > 0.2 ng/m^3^) in non-proximal farmworker households. All indoor AZM air samples in non-farmworker households were < LOD.

### Indoor/Outdoor Concentration Ratios

We present the mean indoor/outdoor ratios by household type in Table 4. All households reported CPF and CPF-O indoor/outdoor ratios < 1, except for one proximal farmworker household that reported a CPF indoor/outdoor ratio of 1.3. The overall indoor/outdoor ratio during spring was 0.17 and 0.05 for CPF and CPF-O, respectively. This indicated higher concentrations outdoors as compared to indoors. Farmworker households reported higher indoor/outdoor ratios of CPF than non-farmworker households (Kruskal–Wallis test, *p* < 0.001).

**Table 4:** Indoor/outdoor air concentration ratios by household type.

Sample type	Proximal farmworker		Proximal non-farmworker		Non-proximal farmworker		Non-proximal non-farmworker		Overall	
	*k*	I/O ratio (mean)	*k*	I/O ratio (mean)	*k*	I/O ratio (mean)	*k*	I/O ratio (mean)	*k*	I/O ratio (mean)
Spring
CPF	6	0.29	2	0.01	7	0.49	5	0.005	20	0.17
CPF-O		0.03^*a*^		0.002^*a*^		0.11^*a*^		0.004^*a*^		0.05
Summer
AZM	6	0.13	2	0.80^*a*^	7	0.77	5	0.24^*a*^	20	0.44
AZM-O		0.48^*a*^		0.67^*a*^		0.79^*a*^		0.72^*a*^		0.72
Winter
CPF^*b*^	2	0.06	1	< LOD (NA)	2	< LOD (NA)	1	< LOD (NA)	6	< LOD (NA)
Note: Indoor/outdoor ratios were calculated individually for each household. The mean ratios were then calculated by proximal farmworker, proximal non-farmworker, non-proximal farmworker, and non-proximal non-farmworker households. I/O, indoor/outdoor ratio: a ratio greater than > 1 indicates higher indoor pesticide concentrations, and a ratio < 1 indicates higher outdoor pesticide concentrations. *k*, number of locations; LOD, limit of detection; NA, not available. ^***a***^These results were calculated using more than 50% substituted values < LOD, therefore they may over or under estimate the true ratio. ^***b***^All other indoor air samples were below the LOD for CPF-O, AZM, and AZM-O, so ratios could not be calculated.										

Most households reported AZM indoor/outdoor ratios less than 1, except for two non-proximal farmworker households that reported indoor/outdoor ratios of 2.1 and 2.5. The overall indoor/outdoor ratio during the summer was 0.44 and 0.72 for AZM and AZM-O, respectively. Many of the reported ratios for CPF-O and AZM-O included substitute values for measurements below the LOD.

### Indoor Surface Deposition

We present the results of surface deposition by household type in Table 5. Surface deposition measurements for CPF ranged from < LOD to 5.7 ng/cm^2^, and 15 of 27 (55%) measurements were < LOD. Surface deposition measurements for AZM were lower than CPF, ranging from < LOD to 1.6 ng/cm^2^, and 7 of 26 (27%) surface deposition measurements were < LOD for AZM. Overall, proximal households had higher levels of CPF on surfaces than non-proximal households (Table 3, Kruskal–Wallis test, *p* < 0.05). We observed very low levels of oxygen analogs in surface deposition samples (all ≤ 0.3 ng/cm^2^). We identified the highest deposition levels of CPF (4 of 27 samples ≥ 1 ng/cm^2^) at two proximal farmworker and two non-proximal farmworker households. We identified the highest deposition levels of AZM (4 of 26 samples ≥ 0.5 ng/cm^2^) at one proximal farmworker and three non-proximal farmworker households. The correlation was stronger for indoor CPF (*R*_s_ = 0.83, *p* < 0.001) than for AZM (*R*_s_ = 0.49, *p* < 0.04). We do not report correlations for oxygen analogs due to the large number of samples below the LOD.

**Table 5:** Summary of indoor surface deposition (ng/cm^2^).

Sample type	Proximal farmworker			Proximal non-farmworker			Non-proximal farmworker			Non-proximal non-farmworker			Total
	*n* (*k*)	*n* < LOD	Mean ± Std (min, max)	*n* (*k*)	*n* < LOD	Mean ± Std (min, max)	*n* (*k*)	*n* < LOD	Mean ± Std (min, max)	*n* (*k*)	*n* < LOD	Mean ± Std (min, max)	*n* (*k*)
Spring
CPF	8 (6)	2	1.7 ± 2.6 (< LOD – 5.7)	4 (2)	2	0.2 ± 0.1 (< LOD – 0.3)	8 (7)	6	0.3 ± 0.6 (< LOD – 1.4)	7 (5)	5	0.1 ± 0.1 (< LOD – 0.3)	27 (20)
CPF-O		3	0.1 ± 0.1 (< LOD – 0.3)		4	< LOD (NA)		6	0.03 ± 0.1 (< LOD – 0.2)		7	< LOD (NA)	
Summer
AZM	8 (6)	2	0.5 ± 0.6 (< LOD – 1.6)	3 (2)	1	0.1 ± 0.1 (< LOD – 0.1)	8 (7)	1	0.3 ± 0.3 (< LOD – 0.7)	7 (5)	3	0.04 ± 0.04 (< LOD – 0.1)	26 (20)
AZM-O		4	0.04 ± 0.1 (< LOD – 0.1)		3	< LOD (NA)		8	< LOD (NA)		7	< LOD (NA)
Winter^*a*^
	3 (2)	3	< LOD (NA)	1 (1)	1	< LOD (NA)	2 (2)	2	< LOD	1 (1)	1	< LOD (NA)	7 (6)
Note: *k*, number of locations; LOD, limit of detection; max, maximum; min, minimum; NA, Not available; *n*, number of samples; *n* < LOD, number of samples < LOD; Std, standard deviation. ^***a***^Winter was a dormant season, therefore a limited number of samples were collected.													

### Winter Season (Control) Results

During the winter, outdoor air concentrations of CPF ranged from < LOD to 5.8 ng/m^3^, and CPF-O ranged from < LOD to 0.4 ng/m^3^ (Table 2). All air samples for AZM and AZM-O were below the LOD. Two proximal farmworker households had detectable indoor air concentrations of CPF ranging from 0.02 to 0.9 ng/m^3^; and all other indoor air samples were below the LOD for CPF-O, AZM, and AZM-O. All indoor surface deposition samples were below the LOD. During the winter, the overall indoor/outdoor ratio for CPF was 0.06.

## Discussion

Our study is the first to use simultaneous passive sampling methods to measure outdoor air concentrations, indoor air concentrations, and surface deposition of OP pesticides and their oxygen analogs in a residential setting. The passive methods captured monthly exposure estimates of CPF, CPF-O, AZM, and AZM-O with agreement between replicate samples and relatively low limits of detection when compared to more traditional active air sampling methods (NIOSH 1994; U.S. EPA 1999). In addition, the passive methods were minimally invasive to research participants. For example, two participants stated that they “hardly noticed the sampler was there.” There is great potential for the use of more passive sampling methods (such as PUF-PAS) in future epidemiology studies, particularly those being conducted in rural areas with limited outdoor electricity. The PUF-PAS provides good means of comparison because larger numbers of samples can be deployed over time, providing useful information for geographical information systems. We found the passive devices to be relatively low cost (e.g., the passive matrices were approximately 1% of the overall cost of daily active air sampling matrices required for the same time period). Since the samplers report cumulative exposures over the course of an entire month, researchers no longer need to exclusively rely on producer application reporting. However, there are some limitations to passive sampling. Since passive samplers report monthly averages, it is not possible to specify ‘peak’ exposure days. We have also found that sampling rates are highly influenced by meteorological factors, such as wind velocity (Armstrong et al. 2014b). In this study, we were able to control for such factors by using depuration compounds, since their rate of loss is also affected by temperature and wind velocity (Tuduri et al. 2006).

Overall, we found that outdoor and indoor air concentrations and surface deposition results for CPF and CPF-O during the spring were 5–10 times higher than AZM and AZM-O during the summer. We continued to measure low levels of airborne CPF and CPF-O (< 6 ng/m^3^) in a subset (*k* = 6) of locations during the dormant winter season. Since the ban on the use of AZM occurred in the year following this study (2012), it is possible that during the summer of 2011 tree fruit producers had already begun to use alternative products. This may have resulted in lower levels of AZM and AZM-O than we expected.

All reported outdoor air concentrations were within the range of concentrations reported in previous studies in California and Washington states (CARB 1998; CDPR 2006, 2009; Fenske et al. 2009). The indoor CPF air concentrations were within or below the range of concentrations reported in a 2004–2005 residential study in New York City conducted by Columbia University (Whyatt et al. 2007). In the present study, the levels of indoor air concentrations of CPF were 0.3–17.5 ng/m^3^, as compared to 0.4–177 ng/m^3^ in the Columbia study. However, CPF was used residentially in New York City for treatment of pests in homes and apartment buildings until 2002. In the present study, CPF was used primarily for outdoor agricultural purposes.

Outdoor air concentrations were higher for households in close proximity to tree fruit fields and households with farmworkers than outdoor concentrations at non-proximal households and non-farmworker households, respectively (Table 3). Proximal households (< 250 m from the nearest tree fruit field) had significantly higher mean outdoor air concentrations of CPF, CPF-O, and AZM (*p* = 0.02, 0.01, and < 0.001, respectively). Various studies have previously demonstrated associations between proximity and higher residential OP pesticide levels in air, dust, and in biomarkers of near-by residents (Loewenherz et al. 1997; Lu et al. 2000; Fenske et al. 2002). However, we defined proximal household by distance (in meters) to only tree fruit fields, and this definition was limited. First, it was unknown if the tree fruit field had been applied with OP pesticides during the sampling period. Second, during the course of the study, we learned that the highest levels of CPF air concentrations were measured at three proximal farmworker households that were within 100 m of corn and wheat fields—in addition to tree fruit fields. In the future, proximity to grain fields should also be considered in geographical regions where corn and grain is more widespread, as CPF is used to control worms, corn borer, and aphid pests in corn and wheat (Gomez 2009).

We found that air concentrations of CPF were lower indoors as compared to outdoors. The trend was similar for AZM, but it was not statistically significant. Inside the home, very little CPF-O or AZM-O was detected. This was expected, as there is less photolysis (via ultraviolet light) to break down parent compounds.

We identified higher indoor air concentrations of CPF in households with close proximity to tree fruit fields (*p* = 0.03) and farmworker status (*p* = 0.01) when compared to households that were non-proximal and did not have farmworkers (Table 3). These findings are similar to other studies that have identified farming households as more contaminated (Simcox et al. 1995; Bradman et al. 1997).

Overall, the indoor/outdoor ratios were lower for CPF than for AZM. During this study, we noted another important factor affecting indoor infiltration. At the end of the study period, the promotora asked household members if they remembered opening the windows during the spring, summer, and winter seasons. During the spring season (while sampling for CPF), only 2 of the households indicated opening windows due to colder weather; whereas during the summer season (while sampling for AZM), 10 of the households indicated opening the windows rather than using air conditioning. During the winter season, no households reported opening windows. The open windows may have contributed to the difference in indoor/outdoor ratios by allowing more AZM to come indoors due to higher air exchange rates (Laumbach et al. 2015). Nevertheless, we found that farmworker households reported higher indoor/outdoor ratios for CPF and CPF-O. Therefore, the potential source of indoor pesticides in non-proximal farmworker households may be more attributable to take home pesticide exposure rather than from outdoor infiltration. To test this theory, future studies should include more factors influencing indoor/outdoor ratios, such as open and closed windows, number of people living in the home, number and type of farmworkers in the home, and type of air conditioning and heating units.

Although indoor surface depositions of CPF and AZM were higher in proximal households than non-proximal households, there were no statistically significant differences observed between farmworker and non-farmworker household deposition samples (Table 3). There was good correlation between indoor surface deposition measurements and air concentrations (*R*_s_ = 0.83 and 0.49 for CPF and AZM, respectively).

There were some limitations to this study. First, we relied on very simple non-parametric statistical test methods rather than multivariable modeling because we were very limited by small sample size. In particular, we found it difficult to identify non-farmworker households that were also proximal since such a large portion of the population is involved in agriculture. Since this was our first attempt to deploy the PUF-PAS samplers for pesticides in a residential setting, we refrained from conducting a larger study in more households. Second, many indoor air and surface deposition samples were below limits of detection, and we had to rely on substituted values for analysis. For future studies using indoor sampling methods for OP pesticides, we suggest using sampling periods of 3–6 months rather than only 1 month. Third, we did not account for the non-independence of replicate samples from the same location and time period, although we deployed replicate samples across all household groups (see Table S1). Finally, although our ideal sampling period was 1 month, the sampling periods ranged from 24 to 32 days, since we had to coordinate the deployment schedule with household members. Although there is variation in pesticide use within a season, it was unlikely that this variation contributed to differences in pesticide levels, as there were no significant differences in sampling deployment periods between household groups.

## Conclusions

We demonstrated the use of passive sampling methods for measuring long-term (1 month) exposures to OP pesticides and oxygen analogs in a remote agricultural area, and encourage others researchers to explore the use of passive sampling devices (like the PUF-PAS) in their region. Exposure data is currently lacking for sub-chronic and chronic epidemiological investigations in rural communities.

We have used passive sampling methods to identify higher outdoor and indoor air concentrations and surface deposition of OP pesticides and their oxygen analogs at both proximal (< 250 m of a tree fruit field) and farmworker households. This study has further confirmed our previous findings on the presence of OP pesticide oxygen analogs in air. On a residential level, human exposures to these oxygen analogs seem to be a greater concern outdoors than indoors. We have found that both proximal and farmworker households have higher levels of exposure to these airborne compounds. When considering cumulative and aggregate effects of human exposure to OP pesticides, the inclusion of oxygen analogs in future risk assessments will be necessary—especially if spending large quantities of time outdoors in rural agricultural areas near applied fields. More research is required to describe the community transport of these pesticide mixtures and how oxygen analogs are formed in outdoor environments.

## Supplemental Material

(136 KB) PDF
