# Supplementary material for: Passive Sampling for Indoor and Outdoor Exposures to Chlorpyrifos, Azinphos-Methyl, and Oxygen Analogs in a Rural Agricultural Community
Source: Environ Health Perspect. 2016 Aug 12;125(3):333–41. doi: 10.1289/EHP425 (PMC5332193; doi:10.1289/EHP425)
Supplement: (136 KB) PDF [file EHP425.s001.acco.pdf]

Environ Health Perspect DOI: 10.1289/EHP425
Advance Publication: Not Copyedited

Note to readers with disabilities: *EHP* strives to ensure that all journal content is accessible to all readers. However, some figures and Supplemental Material published in *EHP* articles may not conform to 508 standards due to the complexity of the information being presented. If you need assistance accessing journal content, please contact ehp508@niehs.nih.gov. Our staff will work with you to assess and meet your accessibility needs within 3 working days.

Supplemental Material

Passive Sampling for Indoor and Outdoor Exposures to Chlorpyrifos, Azinphos-Methyl, and Oxygen Analogs in a Rural Agricultural Community

Jenna L. Gibbs, Michael G. Yost, Maria Negrete, and Richard A. Fenske

Table of Contents

Table S1. Description of replicate passive samples. A replicate sample (duplicate or triplicate) was deployed at all household types during the spring and summer sampling seasons, with the exception of in a non-proximal non-farmworker household during the summer.

Table S1. Description of replicate passive samples. A replicate sample (duplicate or triplicate) was deployed at all household types during the spring and summer sampling seasons, with the exception of in a non-proximal non-farmworker household during the summer.

	Proximal Farmworker	Proximal Non-Farmworker	Non-Proximal Farmworker	Non-Proximal Non-Farmworker	Total
**Spring Outdoor Air Samples**					
Duplicates	✓ (1)	✕	✓ (2)	✓ (2)	5
Triplicates[a]	✓ (2)	✓ (2)	✕	✕	4
Locations (*k*)	7	2	7	7	23
Total Samples (*n*)	12	6	9	9	36
**Spring Indoor Air Samples**					
Duplicates	✓ (2)	✓ (2)	✓ (1)	✕	5
Triplicates[a]	✕	✕	✕	✓ (1)	1
Locations (*k*)	6	2	7	5	20
Total Samples (*n*)	8	4	8	7	27
**Spring Indoor Deposition Samples**					
Duplicates	✓ (2)	✓ (2)	✓ (1)	✕	5
Triplicates[a]	✕	✕	✕	✓ (1)	1
Locations (*k*)	6	2	7	5	20
Total Samples (*n*)	8	4	8	7	27
**Summer Outdoor Air Samples**					
Duplicates	✓ (2)	✓ (2)	✓ (1)	✕	5
Triplicates[a]	✕	✕	✕	✓ (1)	1
Locations (*k*)	7	2	7	7	23
Total Samples (*n*)	9	4	8	9	30
**Summer Indoor Air Samples**					
Duplicates	✓ (2)	✓ (1)	✕	✓ (1)	4
Triplicates[a]	✕	✕	✕	✓ (1)	1
Locations (*k*)	6	2	7	5	20
Total Samples (*n*)	8	3	7	8	26
**Summer Indoor Deposition Samples**					
Duplicates	✓ (2)	✓ (1)	✓ (1)	✕	4
Triplicates[a]	✕	✕	✕	✓ (1)	1
Locations (*k*)	6	2	7	5	20
Total Samples (*n*)	8	3	8	7	26
**Winter Outdoor Air Samples[b]**					
Duplicates	✓ (1)	✕	✕	✕	1
Triplicates[a]	✕	✕	✕	✕	0
Locations (*k*)	2	1	2	1	6
Total Samples (*n*)	3	1	2	1	7
**Winter Indoor Air Samples[b]**					
Duplicates	✓ (1)	✕	✕	✕	1
Triplicates[a]	✕	✕	✕	✕	0
Locations (*k*)	2	1	2	1	6
Total Samples (*n*)	3	1	2	1	7
**Winter Indoor Deposition Samples[b]**					
Duplicates	✓ (1)	✕	✕	✕	1
Triplicates[a]	✕	✕	✕	✕	0

Locations (*k*)	2	1	2	1	6
Total Samples (*n*)	3	1	2	1	7

[a] Fewer triplicate samples were deployed to collect information on variability of the sampling method. All triplicates were deployed at community air monitoring locations, except for one proximal non-farmworker household that allowed the deployment of a triplicate sample in their back yard.

[b] Only one replicate sample was collect in the winter (at a proximal farmworker location indoors and outdoors) since relatively few samples were collected during this dormant season.
